# A Process of Resection-Dependent Nonhomologous End Joining Involving the Goddess Artemis

**DOI:** 10.1016/j.tibs.2017.06.011

**Published:** 2017-09

**Authors:** Markus Löbrich, Penny Jeggo

**Affiliations:** 1 Radiation Biology and DNA Repair, Darmstadt University of Technology, 64287 Darmstadt, Germany; 2 Genome Damage and Stability Centre, School of Life Sciences, University of Sussex, Brighton BN1 9 RQ, UK

**Keywords:** double-strand break repair, nonhomologous end joining, homologous recombination, resection, Artemis

## Abstract

DNA double-strand breaks (DSBs) are a hazardous form of damage that can potentially cause cell death or genomic rearrangements. In mammalian G1- and G2-phase cells, DSBs are repaired with two-component kinetics. In both phases, a fast process uses canonical nonhomologous end joining (c-NHEJ) to repair the majority of DSBs. In G2, slow repair occurs by homologous recombination. The slow repair process in G1 also involves c-NHEJ proteins but additionally requires the nuclease Artemis and DNA end resection. Here, we consider the nature of slow DSB repair in G1 and evaluate factors determining whether DSBs are repaired with fast or slow kinetics. We consider limitations in our current knowledge and present a speculative model for Artemis-dependent c-NHEJ and the environment underlying its usage.

## Trends

A c-NHEJ pathway has been defined involving resection of DSB ends prior to their ligation in G1. Thus, the two main pathways for repairing DSBs in G1 human cells are resection-independent and resection-dependent c-NHEJ.

The resection process in G1 uses many of the same factors used for resection during homologous recombination in G2 but orchestrates them in a manner suited to a c-NHEJ process.

Since Artemis is the only identified factor involved in the resection process whose loss leads to unrepaired DSBs, we refer to this process as Artemis- and resection-dependent c-NHEJ. Loss of other resection factors prevents the initiation of resection but allows resection-independent c-NHEJ.

Artemis- and resection-dependent c-NHEJ makes a major contribution to translocation formation and can lead to previously described microhomology-mediated end joining.

## DNA DSBs Are Repaired by Multiple Pathways

The ability to repair DNA DSBs is a critical determinant of survival to ionising radiation (IR) and other DSB-inducing agents. However, the fidelity with which DSBs undergo repair is also important because inaccurate rejoining can generate genomic rearrangements, potentially leading to carcinogenesis. Therefore, when evaluating DSB repair mechanisms, both rejoining capacity and accuracy should be considered.

**Figure 1:**
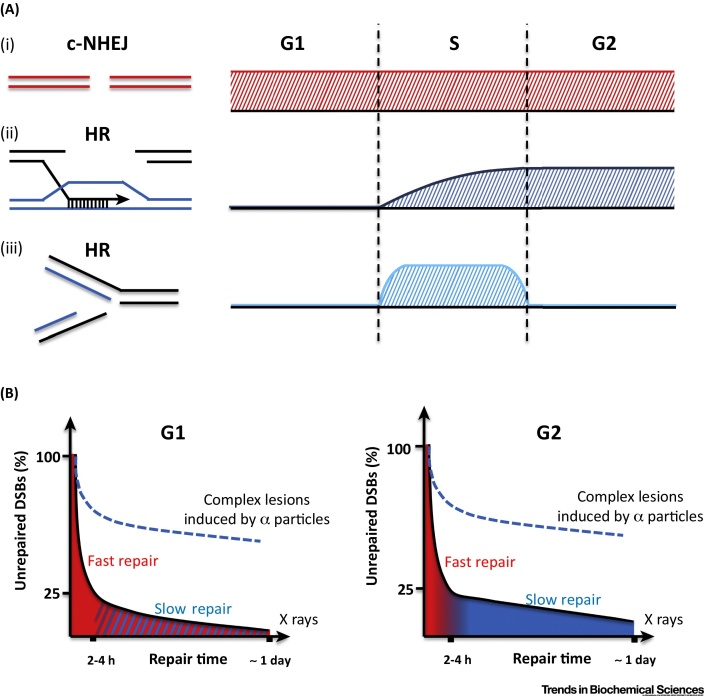
Contribution of NHEJ and HR during the Mammalian Cell Cycle. (A) (i) c-NHEJ represents the major repair pathway for two-ended DSBs in all cell cycle phases. (ii) HR repairs a sub-fraction of two-ended DSBs in G2 and late S phase, when a sister chromatid is present 12, 79. (iii) During S phase, HR also repairs one-ended DSBs that arise following replication fork collapse [80]. The y axes for the panels on the right represent the relative usage of each pathway throughout the cell cycle; it does not reflect the relative usage between the pathways. (B) Most X-ray-induced DSBs in G1 and G2 are repaired with fast kinetics within the first 2–4 h in both cell cycles phases by c-NHEJ (red). However, a subfraction of lesions is repaired with slower kinetics over many hours. This slow component requires ATM and ATM-dependent signalling proteins and is slower in G2 compared with G1 (compare the residual level of unrepaired DSBs at 1 day after X irradiation). Slow DSB repair in G2 is performed by HR (blue). In G1, the slow component represents a form of end joining involving resection and c-NHEJ proteins. This process also requires the nuclease Artemis. Hence, we have called it Artemis-dependent c-NHEJ. Since it has elements of c-NHEJ and HR, it is depicted in red with blue stripes. DSBs induced by calcheomycin or neocarzinostatin are repaired with biphasic kinetics similar to X-ray-induced DSBs. In contrast, following exposure to high linear energy transfer (LET) α particles, the majority of DSBs are repaired with slow kinetics in G1 and G2 (dashed lines) 8, 13, 38. c-NHEJ, canonical nonhomologous end joining; DSB, double-strand break; HR, homologous recombination.

**Canonical nonhomologous end joining** (c-NHEJ, see Glossary) is the predominant repair pathway in all mammalian cell cycle phases for directly induced DSBs, such as those generated by IR. **Homologous recombination** (HR) contributes to the repair of such DSBs in S and G2 phases, although it primarily functions following replication fork stalling or collapse 1, 2. These roles during replication likely represent the essential function of HR and its major contribution to maintaining genomic stability. An overview of the cell-cycle-dependent roles of c-NHEJ and HR is depicted (Figure 1A). Here, we focus on pathways that rejoin directly induced DSBs in G1 or G2 phase.

In addition to c-NHEJ and HR, which have the potential to rejoin DSBs accurately, there is increasing recognition that there are also error-prone pathways involving enzymatically driven resection 3, 4. Alternative NHEJ (alt-NHEJ), which involves DNA ligases I or III, X-ray repair cross-complementing protein (XRCC)1, poly [ADP-ribose] polymerase (PARP)1 and DNA polymerase (Pol) θ 3, 5, 6, represents one such pathway. It exploits microhomology (MH) during rejoining, thereby generating junctional deletions. **Microhomology-mediated end joining** (MMEJ) represents the rejoining of DSBs using small regions of MH [7] and is often taken as being synonymous with alt-NHEJ. However, most studies do not examine the underlying pathway.

Recent work has revealed another process of resection-dependent rejoining, namely resection-dependent c-NHEJ [8]. Here, we define MMEJ as DSB rejoining involving MH regardless of the pathway utilised and discuss the likely contribution of resection-dependent c-NHEJ and alt-NHEJ to MMEJ later in the review. First, we discuss recent insight into the nature of resection-dependent c-NHEJ and present a speculative model for its usage.

## Insight Gained from Evaluating the Kinetics of DSB Repair

Studies using pulsed-field gel electrophoresis (PFGE) or the neutral elution technique have demonstrated that DSBs are repaired with two-component kinetics 9, 10. Enumeration of γH2AX foci in G0/G1 has confirmed that ∼80% of DSBs are repaired rapidly with the remainder being rejoined with substantially slower kinetics [11] (Figure 1B). Both processes, however, require c-NHEJ proteins although the slow process additionally requires the nuclease **Artemis** [11]. Cell-cycle-specific analyses have revealed that G2 cells show similar two-component kinetics, although the slow process in G2 is slower than its G1 counterpart (Figure 1B). Genetic analysis showed that in G2, while the majority of DSBs are repaired with fast kinetics by c-NHEJ, the slow process represents HR [12]. But importantly in this context, the fast and slow repair processes in G0/G1 require c-NHEJ proteins but the slow process additionally requires Artemis.

## The Slow DSB Repair Process in G1 Is Resection Dependent

**Figure 2:**
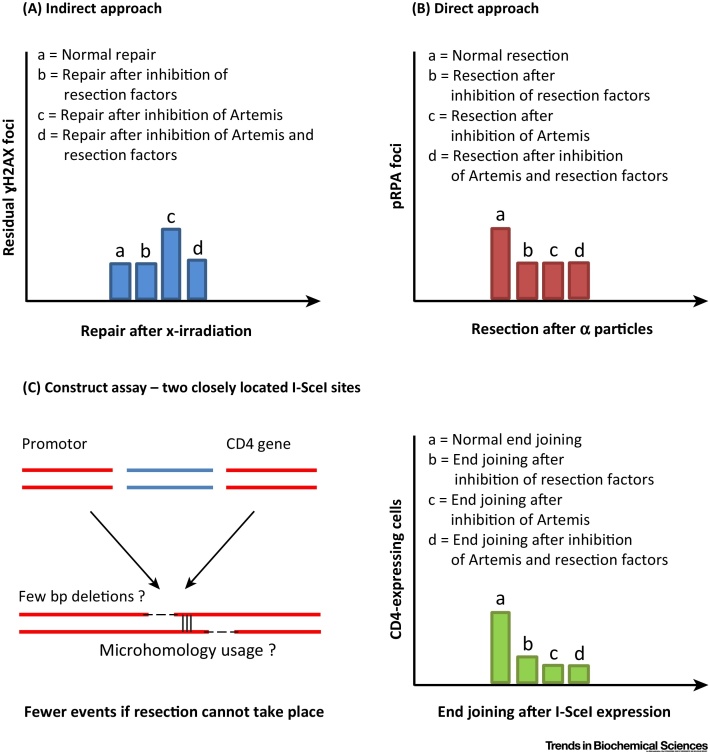
Approaches to Monitor Resection-Dependent c-NHEJ. (A) An indirect, genetic approach assesses rescue of the Artemis repair defect. Artemis deficiency confers a defect in the slow DSB repair component (detected at times >8 h after X irradiation). Loss or inhibition of factors required for resection relieves this repair defect. This reflects a unique role for Artemis in resolving resection intermediates. (B) A direct approach quantifies pRPA foci formation after α-particle irradiation. pRPA foci numbers are reduced following inhibition or depletion of the described resection factors. We note that Artemis deficiency also reduces pRPA foci numbers after such irradiation. (C) An assay involving an integrated construct monitors rejoining of two closely localised I-SceI DSBs. The construct has a promoter and the CD4 gene separated by an intervening sequence (∼3.2 kbp) that prevents transcription of CD4 [14]. Rejoining can occur resulting in loss of the intervening fragment to generate CD4-expressing cells. Such rejoining frequently involves junctional deletions of 1–20 bp and short microhomologies. We have observed diminished rejoining events in Artemis-deficient cells or cells inhibited or depleted for the described resection factors. Thus, loss of the intervening fragment appears to be promoted by resection. All assays are described in [8]. c-NHEJ, canonical nonhomologous end joining; DSB, double-strand breaks; pRPA, phospho-replication protein A.

Given that the slow repair process in G2 (HR) involves resection, recent work examined whether slow c-NHEJ in G1 might also involve DNA end resection; a notion supported by the requirement for Artemis nuclease activity [8]. This model was consolidated using three distinct approaches (Figure 2). An indirect, genetic approach revealed that depletion of several known resection factors does not confer a repair defect after X irradiation in G1 wild-type cells but relieves the repair defect observed in Artemis-deficient cells (Figure 2A). This was interpreted as demonstrating that resection factors function in G1 upstream of Artemis, which cleaves an intermediate structure generated by resection. A direct approach assessed resection by monitoring phospho-replication protein A (pRPA) foci formation after α-particle radiation, when repair also occurs with slow kinetics (Figure 2B) [13]. Finally, a reporter assay developed by the Lopez Laboratory which monitors a process of end joining involving deletions was used to assess factors required for deletion formation. Constructs developed by the Lopez Laboratory monitor rejoining of two close (43 bp) or distant (3.2 kbp) DSBs and such rejoining frequently involves junctional deletions and MH usage 14, 15. **CtIP** (see Glossary) is required for such resection. A similar reporter assay has also shown a requirement for Artemis [16]. Extending this work, it was found that all distant (3.2 kbp apart) DSB-rejoining events involve c-NHEJ and are strictly Artemis dependent [8]. Sequence analysis further revealed that such events frequently ( > 80%) have small junctional deletions and the required factors were characterised.

The findings from these distinct assays were remarkably consistent (Figure 2), supporting the notion that they monitor the same repair process. The findings are interpreted as suggesting that slow DSB repair in G1 is initiated by a resection step involving CtIP, BRCA1, EXO1, EXD2 and **MRE11** exonuclease. If precluded, then repair can occur via c-NHEJ without resection and requirement for Artemis. Significantly, MRE11 endonuclease activity is dispensable for this step, unlike the situation in G2 [17]. It should be noted that although pRPA foci formation can also be observed after high X-ray doses (20 Gy) [8], the same results are obtained after exposure to 2 Gy α particles (Figure 2B). Moreover, the indirect genetic approach exploits 2 Gy X rays (Figure 2A), suggesting that resection-dependent c-NHEJ can arise after physiological doses. A working model for these findings is that Artemis has a downstream role resolving a resection intermediate generated by an upstream step. Thus, Artemis loss confers a repair defect since the initiation step precludes usage of c-NHEJ without resection. Although Artemis-dependent c-NHEJ involves resection, it is unclear if it always leads to end deletions (see discussion below). Resection has been proposed to arise in G1-phase cells but its link to c-NHEJ has not previously been reported 18, 19, 20.

## An Overlapping but Distinct Toolbox for Resection in G1 versus G2

**Figure 3:**
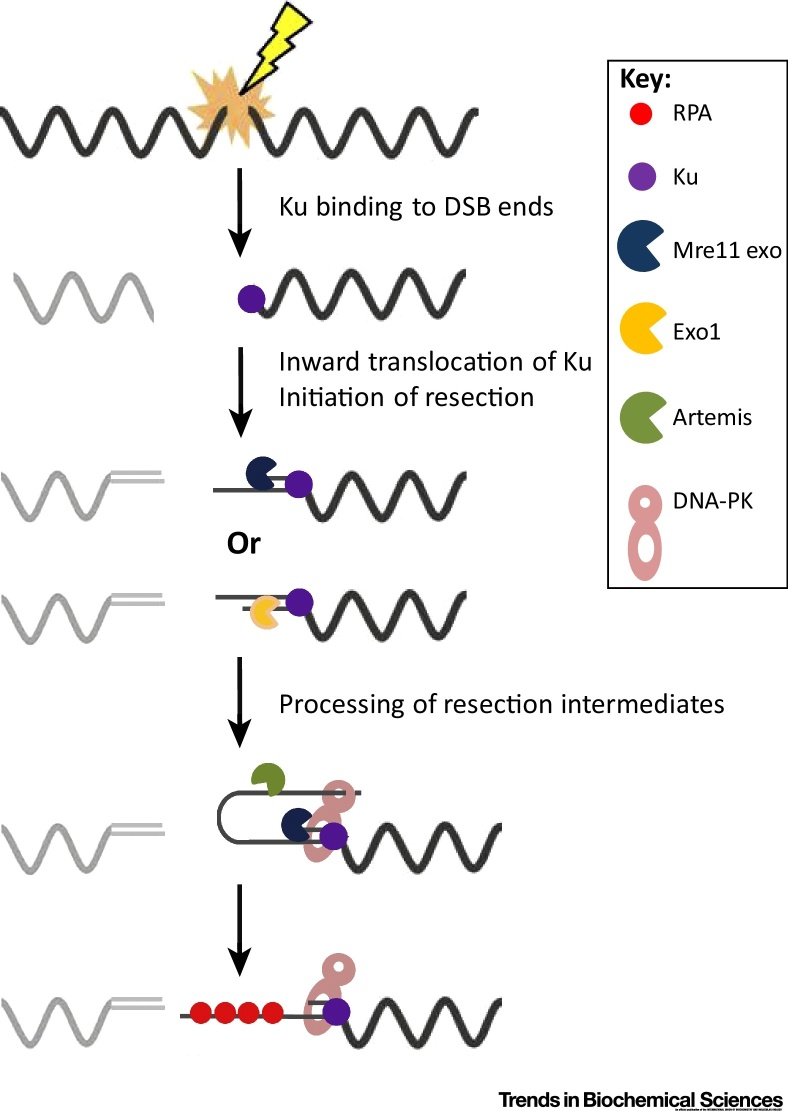
Working Model for the Function of Artemis during Resection-Dependent c-NHEJ. After the initial binding of Ku at the DNA end to promote resection-independent c-NHEJ, Ku translocates further inwards allowing EXO1 or MRE11 exonuclease to resect the 5′ or 3′ end of double-stranded DNA. We raise the possibility that the ss-DNA tail might then be captured by a channel in DNA-PKcs, generating a hairpin intermediate, which requires resolution by Artemis to complete the process. RPA binding to ss-DNA requires Artemis and might be prevented by the hairpin intermediate. c-NHEJ, canonical nonhomologous end joining; DNA-PKcs, DNA-dependent protein kinase catalytic subunit; RPA, phospho-replication protein A; ss-DNA, single-stranded DNA.

Although the slow repair processes in G1 and G2 involve DNA end resection, they are distinct: c-NHEJ in G1 and HR in G2. This is reflected in differences between the resection processes, which suit the cell-cycle-specific repair mechanism, extensive resection for HR in G2, and shorter resection for c-NHEJ in G1 [21]. While the physiological relevance of resection during c-NHEJ in G1 is still unclear, longer resection is likely more beneficial for HR. Ku is well renowned as a resection barrier 22, 23. Interestingly, although Ku80 strongly colocalises with pRPA in G1 cells, it rarely colocalises with RAD51 in G2, consistent with a recent study showing that Ku is lost from longer resected DSBs in G2 concomitant with RAD51 loading 8, 24. This raises the possibility that at later times in G1, Ku remains in the DSB vicinity by inward translocation − a feature of Ku shown in early biochemical studies and consolidated by structural analysis − thereby allowing restricted exonuclease resection at the Ku-vacated ends 25, 26, 27, 28 (Figure 3). However, it is also possible that Ku dynamically releases and rebinds if the single-stranded (ss)-DNA length is short. Lack of CtIP, BRCA1, or the exonucleases may prevent the initiation of resection but allow resection-independent c-NHEJ. In contrast, loss of Artemis appears to allow resection to commence but prevents downstream c-NHEJ usage, conferring a repair defect. In G2, in contrast, resection is initiated internally and progresses bidirectionally via 5′ to 3′ and 3′ to 5′ exonucleases. This mechanism rapidly generates longer ss-DNA regions; a prerequisite for HR and a means to preclude Ku rebinding. Thus, in G2, only loss of CtIP or MRE11 endonuclease activity permits c-NHEJ usage with the exonucleases and BRCA1 functioning downstream of the commitment step.

The kinases regulating resection in G1 versus G2 also differ. In G2, CtIP is constitutively phosphorylated by CDK at Ser327, promoting interaction with BRCA1 [19], while in G1, Ser327 CtIP phosphorylation promoting BRCA1 interaction is Polo-like kinase (PLK)3- and IR-dependent [13]. Depletion or inhibition of PLK3 bypasses the Artemis requirement for DSB repair in G1, consistent with a role in initiating resection, but does not influence HR in G2. ATM directly phosphorylates PLK3, CtIP, BRCA1, and KRAB-associated protein (KAP)-1 and thus can potentially orchestrate resection at multiple levels.

BRCA1 also appears to have an overlapping role in G1 with its function in G2, but there are distinctions that result in more limited resection in G1 versus G2. BRCA1 relieves a 53BP1-dependent barrier to resection in S/G2 via Rap1 interacting factor (RIF)1 and/or Pax transactivation domain-interacting protein (PTIP) 29, 30, 31, 32, 33. Consequently, combined loss of BRCA1 and 53BP1 enables resection and DSB repair, although it occurs by single-strand annealing rather than gene conversion [4]. Similarly, BRCA1 relieves a 53BP1 barrier to resection in G1 [8]. Repositioning of 53BP1 in G2 is detectable by high-resolution imaging but this is not observed in G1; likely due to a more limited degree of repositioning [34]. Possibly related to this finding, BRCA1 is expressed at lower levels in G1 versus G2.

## Working Model for the Function of Artemis

Artemis is an endonuclease that cleaves a hairpin intermediate during V(D)J recombination [35]. Artemis also has exo- and endonuclease activity, leading to proposals that it functions during DSB end processing [36]. Significantly, Artemis nuclease activity is dependent on DNA-dependent protein kinase, catalytic subunit (DNA-PKcs) autophosphorylation, suggesting that DNA-PK may remodel the end to allow Artemis cleavage 35, 37. Importantly, loss of Artemis activity only confers a defect in the slow DSB repair process in G1 but siRNA depletion of resection factors bypasses the need for Artemis, suggesting that Artemis functions downstream of resection at a stage that precludes the usage of c-NHEJ without resection 8, 11. We propose that following Ku vacation of DSB ends by inward translocation (as proposed above), exonuclease-mediated end-resection takes place in either a 5′ or 3′ direction and in distinction to the endonucleolyic incision model proposed for G2 [38]. As a speculative model, we propose that the ss-DNA ends might then be captured by a channel in DNA-PKcs identified by structural studies and of a size that can accommodate ss- but not double-stranded (ds)-DNA 39, 40 (Figure 3). This might create a hairpin intermediate, the known Artemis substrate during V(D)J recombination. Significantly, this model is consistent with the key structural features for Artemis cleavage derived from substrate analysis and with the need for DNA-PK to remodel the DNA ends for Artemis cleavage 37, 41. Notably, however, Artemis loss diminishes pRPA foci after high X-ray doses and high linear energy transfer (LET) radiation [8]. This shows that ss-DNA must become available for RPA binding only after Artemis cleavage, suggesting that the hairpin structure precludes RPA binding to ss-DNA (Figure 3). The fact that pRPA foci are only observed after high X-ray doses or α particles suggests that longer resection may ensue in these situations.

## Contribution of Artemis-Dependent c-NHEJ to Translocations

**Figure 4:**
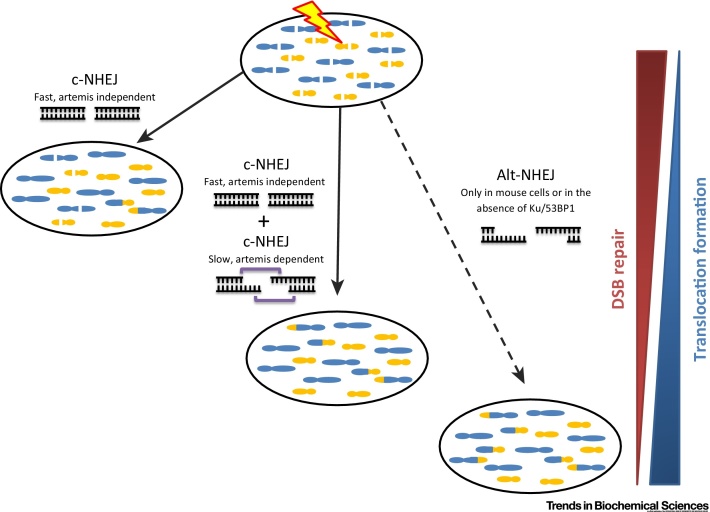
Propensity for DSB Repair Processes in G1 Phase to Give Rise to Translocations. Repair of DSBs in G1 by end-joining mechanisms can give rise to translocations when the incorrect break ends are mis-rejoined. However, the propensity for the different end-joining processes to produce translocations differs. Compared with resection-independent c-NHEJ, which repairs the majority of X-ray-induced DSBs with fast kinetics, Artemis-dependent c-NHEJ has ∼4-fold higher potential to cause translocations after 7 Gy [8]. We stress that translocations arise with dose in a linear-quadratic manner and that the relative contribution of the fast and slow repair processes may depend on dose. This increased propensity for translocation formation is likely the result of resection since preventing the initiation of resection by siRNA-mediated CtIP depletion significantly decreases translocation formation [13]. We speculate that DSBs undergoing resection are normally stabilized by bridging factors (depicted as purple brackets) whose occasional release from the ends can cause translocations. Chromatin compaction factors and/or DNA-PK bound to the ends may serve as such bridging factors. Alt-NHEJ processes, which do not seem to make an appreciable contribution to DSB repair in G0/G1-phase human cells (and are hence displayed by a dashed line), have a high potential to cause translocations in mouse cells. The figure shows all DSBs being repaired by alt-NHEJ since we do not know the contribution of alt-NHEJ versus c-NHEJ in mouse cells. The relative contributions of the three processes for DSB repair and the propensity for translocation formation are shown on the right. Alt-NHEJ, alternative nonhomologous end joining; c-NHEJ, canonical nonhomologous end joining; DNA-PK, DNA-dependent protein kinase; DSB, double-strand break.

Translocation formation, namely rejoining of the wrong DSB ends, can promote carcinogenesis [42]. Recent studies monitoring translocation of programmed and endogenously arising DSBs to defined bait DSBs have shown that they can arise via c-NHEJ and alt-NHEJ 43, 44, 45. Here, we evaluate specifically the origin of translocations arising in G0/G1-phase cells and the relative contribution of the fast and slow DSB repair process. To focus on translocations that arise specifically in G0/G1 phase, Biehs *et al*. exploited a well-characterised procedure involving fusion of G1 to mitotic cells that promotes premature chromosome condensation in G1 cells. Since mitotic cells are downregulated for c-NHEJ and since fusion takes place only at the end of the repair period, repair processing occurs predominantly in G1 cells prior to their fusion [46]. Significantly, it was shown that although Artemis-dependent c-NHEJ repairs only 20% of the induced DSBs, it generates half of the translocations after 7 Gy X rays [8] (Figure 4). Importantly, alt-NHEJ, which significantly contributes to translocation formation in mice, does not influence G1 translocations arising in human cells unless Ku or 53BP1 are absent 5, 18, 47. The inability to detect any contribution of alt-NHEJ in human cells could be a consequence of their greatly increased levels of Ku and DNA-PK activity 48, 49. Assessment of the relative contribution of the two resection processes to translocation formation in G1 mouse versus human cells, however, requires a comparative analysis of translocations in G1 cells, which has not yet been successfully undertaken (see Outstanding Questions). The technique used above and the discussion addresses the origin of translocations arising in irradiated G0/G1 phase cells. We stress, however, that translocations can also arise during replication and potentially via aberrant rejoining events in S and G2 phase.

## Chromatin Environment and Damage Complexity Influence Pathway Usage

**Figure 5:**
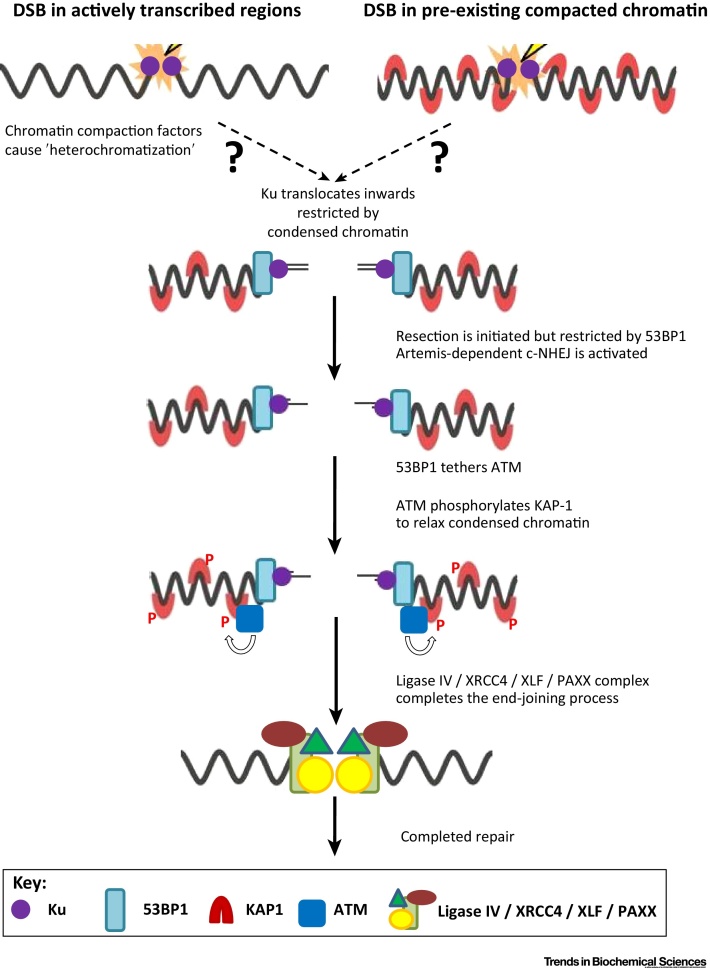
Key Figure: Artemis-Dependent c-NHEJ within the Chromatin Environment

Which features determine the usage of the slow repair pathways versus fast c-NHEJ? (See Outstanding Questions.) An expectation was that DNA damage complexity would be a determining factor. However, exposure to X rays, neocarzinostatin or tert-butyl hydroperoxide confers a similar magnitude of the slow component, although the latter two agents generate DSBs with homogeneous end structures 50, 51. Moreover, unrepaired DSBs accumulate in Artemis-deficient fibroblasts maintained under nonreplicating conditions, suggesting that Artemis also functions in repairing DSBs arising from endogenous oxidative damage [51]. Additionally, the magnitude of the slow component (∼20% of induced DSBs) is dose independent over a large dose range, inconsistent with a model of limiting repair capacity [52]. This raises the possibility that for these agents the structural and functional properties of the chromatin environment rather than the nature of the DSB might determine repair pathway usage. Furthermore, it is noteworthy that the slow repair process requires ATM and ATM signalling factors, including 53BP1, and that depletion of chromatin compacting factors including KAP-1, heterochromatin protein (HP)1α/β or SETDB1 bypasses the need for ATM and 53BP1 for the slow repair component 53, 54. ATM phosphorylates KAP-1 on Ser824 and expression of nonphosphorylatable S824A KAP-1 confers a DSB repair defect even in the presence of ATM, while phospho-mimic S824D KAP-1 bypasses the need for ATM [50]. 53BP1 is required for defined ATM and phospho-KAP-1 foci at DSB sites, which has been proposed to promote local chromatin decompaction [55]. Based on these and additional findings, it was proposed that slow DSB repair represents the repair of DSBs arising within pre-existing heterochromatin (designated HC-DSBs), which represents ∼20% of genomic DNA (Figure 5, Key Figure, right panel). However, there is increasing evidence that compacting factors are recruited to DSBs, enhancing compaction in the DSB vicinity and promoting HR 56, 57, 58, 59, 60, 61. Thus, we suggest the alternative possibility that ATM serves to relax compacted chromatin arising during repair rather than to promote the repair of DSBs that arise within pre-existing HC (Figure 5).

However, IR uniquely induces complex DSBs with multiple lesions in close proximity, which also influences the kinetics of DSB repair 62, 63, 64. X rays are low-LET radiation, which generate DSBs of modest complexity. In contrast, high-LET radiation, such as heavy ions or α particles, induces highly complex DSBs 65, 66. Significantly, most DSBs induced by high-LET radiation are repaired with similar kinetics to the X-ray-induced slow DSB repair component 38, 67 (Figure 1B). Furthermore, in G2, an increased fraction of high-LET-induced DSBs undergo resection and repair by HR compared to X-ray-induced DSBs [38]. Thus, high-LET-induced DSBs undergo resection and repair by HR even in genomic locations where fast DSB repair without resection normally prevails, suggesting that the propensity for high-LET-induced DSBs to undergo repair by the slow process (in G1 and G2) is not determined by the structure and/or functionality of the chromatin environment but rather by DSB complexity. Hence, both DNA damage complexity and genomic location can influence DSB repair kinetics, which itself reflects repair pathway usage. After X rays, the genomic location may be the major determining factor but after high-LET radiation, the damage complexity exerts more influence.

## A Novel Model for Artemis-Dependent c-NHEJ and Pathway Usage

In addition to translocation formation, another aspect of DSB repair fidelity is junctional accuracy, namely the generation of junctional deletions or insertions (see Outstanding Questions). The model involving Artemis-dependent cleavage of a hairpin intermediate demands that small deletions arise frequently during Artemis-dependent c-NHEJ, which appears unlikely if one considers the large amount of small deletions that would arise. Thus, full insight into the resection-dependent repair mechanism including an understanding of how the resected ends are rejoined may still be lacking. As a speculative model we propose that an RNA template with homology to the DSB site could be exploited to reconstitute sequence information. There has been increasing evidence that RNAs function during DSB repair, with most studies focusing on HR, either to regulate resection or to provide a repair template 68, 69, 70, 71. A seminal paper provided proof of principle that RNA could serve as a template for DSB repair in yeast [72]. While the RNA could arise via normal transcription, recent studies have provided evidence for damage response RNAs arising from the DSB end sequences in a DROSHA- and DICER-dependent manner 73, 74. Additionally, RNA Pol II is recruited to ss-DNA generated by DSB resection and correlates with DNA:RNA hybrid formation [71]. Intriguingly, older studies have shown that RNA Pol II can initiate transcription without other components of the transcription machinery from a 3′-OH DNA end [75]. Further studies should explore this speculative proposal (see Outstanding Questions).

Important recent studies exploiting AsiSI-induced DSBs have provided evidence that HR factors associate with DSBs in actively transcribed regions and that such DSBs undergo clustering and delayed repair in G1 76, 77. Given that there is a similar magnitude of the slow repair process in G1 versus G2 after IR [12], it appears likely that HR and resection-dependent c-NHEJ repair the same class of DSBs, raising the possibility that the subset of DSBs undergoing resection-dependent c-NHEJ could represent or encompass those in actively transcribed regions. If this is the case, then the fidelity of resection-dependent c-NHEJ is important to consider. An intriguing possibility raised by the model that RNA serves as a template during the slow repair process is that such DSBs could actually represent those in transcriptionally active regions (which may be those with preformed transcription-dependent RNA or an open structure allowing end-templated RNA transcription). Since ATM has a role in relaxing compacted chromatin during slow DSB repair, we suggest that the chromatin surrounding these DSBs becomes compacted during repair, with its subsequent relaxation being a prerequisite for the completion of repair as discussed above (Figure 5). The fact that the slowly repairing X-ray-induced DSBs undergo resection, despite their ability to be repaired without resection, adds to the notion that resection-dependent end joining may not predominantly form deletions. Indeed, usage of a resection-dependent process where repair without resection is equally possible argues in favour of a model presenting the best-possible solution (an accurate resection-dependent process) for the more important genomic regions (transcribed genes).

In summary, the nature of the DSBs repaired with slow kinetics remains unclear. Given that recent studies have proposed that DSBs within actively transcribed regions persist at later times 76, 77, we speculate that the slow DSB repair process after X irradiation may encompass the repair of DSBs within transcribed regions, as well as the repair of DSBs within HC, as previously proposed. Furthermore, to enhance the accuracy of rejoining, we raise the possibility that the process could use end- or transcript-derived RNA as a template to minimise deletion formation. Thus, rather than reflecting an inaccurate process involving microhomologies and frequent deletions, the process may efficiently promote accurate junctional reconstitution. However, it appears that this arises at the cost of rare translocation formation.

## MMEJ, alt-NHEJ, and Artemis-Dependent c-NHEJ: Ménage à Trois

Earlier in this review, we defined MMEJ as any process involving MH usage, rather than representing a specific repair process. Both resection-dependent c-NHEJ and alt-NHEJ could represent the mechanism underlying MMEJ. Importantly, we have observed that loss of alt-NHEJ proteins does not impact upon DSB rejoining or translocation formation in G0/G1 phase primary human cells. Thus, we propose that MMEJ in normal human cells arises predominantly via c-NHEJ and represents errors that arise during Artemis- and resection-dependent c-NHEJ; for example, if an RNA template is unavailable. This might occur at complex DSBs induced in nontranscribed regions, at DSBs in heterochromatic regions, or during mis-rejoining of previously unconnected DSB ends. However, certain cancer cells may specifically promote alt-NHEJ to gain genomic instability.

## Concluding Remarks

DSB repair occurs by fast and slow processes in G0/G1 cells with specific factors being required for the slow process. HR represents the slow rejoining process in G2 phase. Here, we discuss recent insight showing that the slow DSB repair mechanism in G1 represents Artemis- and resection-dependent c-NHEJ. Thus, resection lies at the centre stage determining pathway usage. However, although the toolbox of nucleases that promote resection in the two cell cycle phases is similar, there are important distinctions. In G2, MRE11 endonuclease activity promotes extensive resection demanding repair by HR, while in G1, a distinct process progresses more limited resection and repair by Artemis-dependent c-NHEJ. The slow process has hitherto been argued to represent the repair of DSBs within pre-existing HC. Based on recent findings, we suggest that it may in fact represent the repair of DSBs in open chromatin, which become compacted and subsequently decompacted during repair. Additionally, we propose a speculative model that the slow repair process may involve an RNA template to prevent junctional deletions.

Outstanding Questions What is the nature of the DSBs that undergo Artemis-dependent c-NHEJ? Are they breaks induced within pre-existing compacted DNA regions or regions opened for transcription, which become compacted during repair?

What is the fidelity of the fast and slow DSB repair processes in G1 phase? How frequently do these processes generate deletions? Does resection enhance the potential for deletions or can resected ends utilize a template for repair to limit deletion formation or is accuracy maintained in some other way?

How is Ku binding to DSB ends regulated during resection in G1? Does Ku remain bound to ends while translocating inwards during resection, or is it transiently released and re-bound to resected ends? Is the presence and movement of Ku and/or 53BP1 regulating the extent of resection?

How does Artemis function downstream of the initiation of resection? The results suggest that it processes repair intermediates, which arise from the resection process. What is the nature of these intermediates and why do they specifically require Artemis for their resolution?

How does chromatin remodelling affect resection in G1? While chromatin compaction and ATM-mediated chromatin relaxation might serve to transiently stabilize DSB ends during resection, additional chromatin remodelling at the DSB ends is likely required to allow resection. This has been studied for HR in G2 but not for Artemis-dependent c-NHEJ in G1.

## Glossary

**Artemis**: Protein whose deficiency leads to a form of radiosensitive severe combined immunodeficiency. It is often described as a c-NHEJ component, although it is only essential for a subfraction of DSB end joining. Artemis has nuclease activity, and a β-CASP and β-lactamase domain. Since it is hitherto the only identified factor whose loss leads to unrepaired DSBs specifically during resection-dependent c-NHEJ, we have called the process Artemis and resection dependent c-NHEJ.
**BRCA1/2**: BRCA1 and 2 are frequently mutated in forms of hereditary breast cancer. BRCA2 functions in HR by aiding the loading of RAD51. The function of BRCA1 is less clear but it has roles during HR and Artemis-dependent c-NHEJ with the available evidence suggesting that it counteracts the anti-resection function of 53BP1.
**Canonical non-homologous end-joining (c-NHEJ)**: An end-joining DSB repair pathway that involves the initial binding of the Ku heterodimer to the DNA end. Then the DNA-dependent protein kinase catalytic subunit DNA-PKcs is recruited, generating the DNA-PK complex. Finally, a complex of DNA ligase IV, XRCC4, XLF, and PAXX carries out the rejoining step.
**CtIP**: Is the mammalian homologue of the yeast protein Sae2 and is required for resection in mammalian cells. It associates with the MRN complex.
**Microhomology-mediated end-joining (MMEJ)**: MMEJ is often described as being synonymous with alt-NHEJ. However, most studies examining MMEJ do not assess the rejoining process. Here, we define MMEJ as representing a form of rejoining that exploits a short region of microhomology, which is inherently erroneous due to small deletions. However, since alt-NHEJ does not contribute significantly to DSB rejoining in G0/G1-phase human cells, we discuss here the likelihood that most MMEJ in G0/G1-phase human cells occurs by Artemis-dependent c-NHEJ.
**Homologous recombination (HR)**: A DSB rejoining pathway that uses extensive regions of homology and information from a homologous template. In mammalian cells, the sister chromatid provides the source of homology, therefore, HR only occurs in late S and G2 phase, after the sister chromatid has been synthesised. The process involves the generation of ss-DNA by end resection, which is rapidly bound by RPA. RAD51 then replaces RPA by a process involving BRCA2. This leads to strand exchange and the formation of heteroduplex DNA. Finally, the region encompassing the DSB is recovered using the undamaged strand as a template for repair synthesis and resolution or dissolution occurs, generating crossover or non-crossover events.
**MRE11**: MRE11 is part of the MRN complex, which encompasses three distinct proteins, MRE11, RAD50, and NBS1. The complex is an important sensor of DSBs and facilitates ATM recruitment to DSBs and ATM activation. MRE11 has exonuclease and weak endonuclease activity. In yeast, Mre11 endonuclease activity is stimulated by Sae2, the homologue of CtIP [78]. MRE11 endonuclease activity is only required for HR; MRE11 exonuclease activity is required for HR and Artemis-dependent c-NHEJ.
